# Evaluation of Innotrac Aio! Second-Generation Cardiac Troponin I Assay: The Main Characteristics for Routine Clinical Use

**DOI:** 10.1155/JAMMC/2006/39325

**Published:** 2006

**Authors:** P. Hedberg, J. Valkama, E. Suvanto, S. Pikkujämsä, K. Ylitalo, E. Alasaarela, M. Puukka

**Affiliations:** 1 Laboratory of Oulu University Hospital, Department of Clinical Chemistry University of Oulu P.O. Box 500 Oulu 90029 Finland; 2 Emergency Unit Department of Internal Medicine Oulu University Hospital Oulu 90029 Finland; 3 Kuusamo Hospital Laboratory Kuusamo 93600 Finland; 4 Division of Nephrology Department of Internal Medicine Oulu University Hospital Oulu 90029 Finland; 5 Division of Cardiology Department of Internal Medicine Oulu University Hospital Oulu 90029 Finland; 6 Division of Rheumatology Department of Internal Medicine Oulu University Hospital Oulu 90029 Finland

## Abstract

The availability of a simple, sensitive, and rapid test using whole blood to facilitate processing and to reduce the turnaround time could improve the management of patients presenting with chest pain. The aim of this study was an evaluation of the Innotrac Aio! second-generation cardiac troponin I (cTnI) assay. The Innotrac Aio! second-generation cTnI assay was compared with the Abbott AxSYM first-generation cTnI, Beckman Access AccuTnI, and Innotrac Aio! first-generation cTnI assays. We studied serum samples from 15 patients with positive rheumatoid factor but with no indication of myocardial infarction (MI). Additionally, the stability of the sample with different matrices and the influence of hemodialysis on the cTnI concentration were evaluated. Within-assay CVs were 3.2%–10.9%, and between-assay precision ranged from 4.0% to 17.2% for cTnI. The functional sensitivity (CV = 20 %) and the concentration giving CV of 10% were approximated to be 0.02 and 0.04, respectively. The assay was found to be linear within the tested range of 0.063–111.6 μ g/L. The correlations between the second-generation Innotrac Aio!, Access, and AxSYM cTnI assays were good (*r* coefficients 0.947–0.966), but involved differences in the measured concentrations, and the biases were highest with cTnI at low concentrations. The second-generation Innotrac Aio! cTnI assay was found to be superior to the first-generation assay with regard to precision in the low concentration range. The stability of the cTnI level was best in the serum, lithium-heparin plasma, and lithium-heparin whole blood samples (*n* = 10 , decrease < 10 % in 24 hours at  +20^°^C and at  +4^°^C. There was no remarkable influence of hemodialysis on the cTnI release. False-positive cTnI values occurred in the presence of very high rheumatoid factor values, that is, over 3000 U/L. The 99th percentile of the apparently healthy reference group was  ≤ 0.03 μ g/L. The results demonstrate the very good analytical performance of the second-generation Innotrac Aio! cTnI assay.


Hindawi Publishing Corporation
Journal of Automated Methods and Management in Chemistry
Volume 2006, Article ID 39325, Pages 1–7
DOI 10.1155/JAMMC/2006/39325

### Evaluation of Innotrac Aio! Second-Generation Cardiac Troponin I Assay: The Main Characteristics for Routine Clinical Use

**P. Hedberg,[1] J. Valkama,[2] E. Suvanto,[3] S. Pikkujämsä,[4] K. Ylitalo,[5] E. Alasaarela,[6] and M. Puukka[1]**

[1] *Laboratory of Oulu University Hospital, Department of Clinical Chemistry, University of Oulu, P.O. Box 500, 90029 Oulu, Finland*
[2] *Emergency Unit, Department of Internal Medicine, Oulu University Hospital, 90029 Oulu, Finland*
[3] *Kuusamo Hospital Laboratory, 93600 Kuusamo, Finland*
[4] *Division of Nephrology, Department of Internal Medicine, Oulu University Hospital, 90029 Oulu, Finland*
[5] *Division of Cardiology, Department of Internal Medicine, Oulu University Hospital, 90029 Oulu, Finland*
[6] *Division of Rheumatology, Department of Internal Medicine, Oulu University Hospital, 90029 Oulu, Finland*

Received 30 October 2005; Revised 7 February 2006; Accepted 13 February 2006

The availability of a simple, sensitive, and rapid test using whole blood to facilitate processing and to reduce the turnaround time could improve the management of patients presenting with chest pain. The aim of this study was an evaluation of the Innotrac Aio! second-generation cardiac troponin I (cTnI) assay. The Innotrac Aio! second-generation cTnI assay was compared with the Abbott AxSYM first-generation cTnI, Beckman Access AccuTnI, and Innotrac Aio! first-generation cTnI assays. We studied serum samples from 15 patients with positive rheumatoid factor but with no indication of myocardial infarction (MI). Additionally, the stability of the sample with different matrices and the influence of hemodialysis on the cTnI concentration were evaluated. Within-assay CVs were 3.2%–10.9%, and between-assay precision ranged from 4.0% to 17.2% for cTnI. The functional sensitivity ($CV = 20\%$) and the concentration giving CV of 10% were approximated to be 0.02 and 0.04, respectively. The assay was found to be linear within the tested range of 0.063–111.6 $\mu$g/L. The correlations between the second-generation Innotrac Aio!, Access, and AxSYM cTnI assays were good ($r$ coefficients 0.947–0.966), but involved differences in the measured concentrations, and the biases were highest with cTnI at low concentrations. The second-generation Innotrac Aio! cTnI assay was found to be superior to the first-generation assay with regard to precision in the low concentration range. The stability of the cTnI level was best in the serum, lithium-heparin plasma, and lithium-heparin whole blood samples ($n = 10$, decrease $< 10\%$ in 24 hours at $+20^\circ$C and at $+4^\circ$C). There was no remarkable influence of hemodialysis on the cTnI release. False-positive cTnI values occurred in the presence of very high rheumatoid factor values, that is, over 3000 U/L. The 99th percentile of the apparently healthy reference group was $\leq 0.03$ $\mu$g/L. The results demonstrate the very good analytical performance of the second-generation Innotrac Aio! cTnI assay.

Copyright © 2006 P. Hedberg et al. This is an open access article distributed under the Creative Commons Attribution License, which permits unrestricted use, distribution, and reproduction in any medium, provided the original work is properly cited.

### 1. INTRODUCTION

Cardiac troponin I cTnI is a sensitive and specific marker of acute coronary syndromes and myocardial damage. During the past few years, it has become the most preferred biochemical marker of myocardial infarction. The consensus document of the Joint European Society of Cardiology/American College of Cardiology (ESC/ACC) and the American Heart Association (AHA) gives specific recommendations concerning the use of biomarkers for the detection of myocardial infarction [1, 2]. To avoid misclassification arising from assay imprecision, the consensus committee proposed that the 99th percentile reference limit should be measured with total imprecision (CV) $< 10\%$. The purpose of the current study was to evaluate the performance of the second-generation cardiac troponin I assay on the Innotrac Aio! immunoassay platform, to establish the cutoff limit, and to investigate cTnI release in rheumatoid arthritis and the influence of hemodialysis on cTnI release.

### 2. MATERIALS AND METHODS

### 2.1. *Samples*

Lithium-heparin plasma samples (Terumo Venoject Lithium Heparin, Cat. no. VP 050SHL, Terumo Europe N. V. Leuven,

Belgium, with a total heparin concentration of 15.9 IU/mL of whole blood) for cTnI determinations were randomly collected from the routine samples of chest pain patients and from apparently healthy persons. For a comparison of whole blood and plasma samples, an additional lithium-heparin sample was taken for whole blood determination in parallel with the routine plasma sample. For the 99th percentile determination of healthy volunteers with different sample matrices, a lithium-heparin tube, an additional K2-EDTA anticoagulated tube (Becton Dickinson Vacutainer, Cat. no. 368856, Becton Dickinson Systems, Plymouth, UK), and a serum tube (Terumo Venoject II, Cat. no. VP-050SPZ, Terumo Europe NV, Leuven, Belgium) were taken. EDTA plasma samples and serum samples were used for the hemodialysis and rheumatoid arthritis studies, respectively. In order to obtain the lithium-heparin plasma, serum, or EDTA plasma samples, the whole blood tubes were centrifuged at 2000 g for 15 minutes. The supernatant plasma, serum, and whole blood were either analyzed immediately or stored at 4°C for analysis within 8 h. For long-term storage, the plasma and serum samples were placed at −20°C. Before analysis, the frozen samples were thawed at room temperature, mixed, and centrifuged to remove any particulate material.

All the patients granted permission for additional blood samples to be collected, and the local hospital ethics committee approved the study.

### 2.2. Analytical methods cTnI analyses were made using the Innotrac Aio! analyzer (Innotrac Diagnostics Oy, Turku, Finland), and comparative analyses were made with the Abbott AxSYM system (Abbott Laboratories, Diagnostic Division, Abbott Park, Ill, USA) and Beckman Access (Beckman Coulter, Inc., Chaska, Minn). The rheumatoid factor determinations were measured with Hitachi 911 (Roche Diagnostics GmbH, D-68298 Mannheim). The Hct of the whole blood samples was determined by Abbott Cell-Dyn 4000 (Abbott Laboratories, Diagnostic Division, Abbott Park, Ill, USA).

The Innotrac Aio! analyzer is a fully automated random-access immunoanalyzer based on a universal all-in-one dry-reagent concept [3, 4]. The physical dimensions of the satellite version are weight 65 kg, height 650 mm, width 620 mm, and depth 500 mm. The central version with the sample conveyor has a weight of 80.5 kg and width of 875 mm. All analyte-specific reagents are dry-coated onto the bottom of the analyte cups, which are packed into analyte pens (12 cups/pen), and only the addition of the sample and the generic assay buffer is required. To perform the assay, the sample and the buffer are, under continuous shaking, washed and dried, after which the signal of intrinsically fluorescent Europium lanthanide chelate is read from the dry surface. The quantitative results are available within 18 minutes after the addition of the sample. Calibration of the assays is done whenever a new kit lot is taken into use. Each kit box contains a bar code for reading the factory-defined calibration data, which are used as a lot-specific reference curve. When the instrument detects analyte pens with a new lot number, it requests the system to load a corresponding calibration pen. The calibration is then performed automatically.

### 2.3. Imprecision

Three whole blood and 3 plasma samples with variable cTnI concentrations were used to test within-assay precision ($n$ = 20 per run). Commercial controls (Quantimetrix Cardiasure, levels 1, 2, and 3, Quantimetrix Corporation, Redondo Beach, Calif, USA) and a low serum pool were used to test interassay precision. Interassay precision was determined by analyzing duplicate control samples twice a day on 10 separate days ($n = 40$).

### 2.4. Linearity

Dilution linearity was investigated by serial dilution of five samples of known cTnI concentrations. All the samples were diluted with the instrument buffer solution.

### 2.5. Comparison of sample materials and methods

The first comparative analyses of second-generation Innotrac Aio! cTnI assay, Beckman Access AccuTnI, and Abbott AxSYM cTnI (first-generation) were carried out with a set of 91 lithium-heparin plasma samples. Lithium-heparin whole blood, lithium-heparin plasma, and serum comparisons were carried out with 38 patient samples and the Aio! analyzer. The hematocrit (Hct) value of each lithium-heparin whole blood sample was determined by Cell-Dyn 4000. The second comparative analyses of the Innotrac Aio! first-generation, Innotrac Aio! second-generation, and AxSYM cTnI assays were carried out with a total of 97 lithium-heparin plasma samples.

### 2.6. Detection limit, functional sensitivity, and the concentration giving CV of 10%

To determine the lowest measurable concentration of the Innotrac Aio! second-generation cTnI that could be distinguished from zero, 20 replicates of the zero calibrator were analyzed. The detection limits were calculated by determining the mean concentration plus 3SD. Functional sensitivity (20% CV) and the concentration giving a 10% CV result were determined by analyzing lithium-heparin plasma pools serially diluted with instrument buffer 1-2 per day in duplicates for 10 days ($n = 30$). The interassay precision value obtained using a serum pool was also used for the determination of 10% and 20% CVs.

### 2.7. 99th percentile reference limits

Samples for the 99th percentile determination were collected from an apparently healthy reference group (median ages 43 and 44, and ranges 22–59 and 22–67 years for women and men, resp.). The determinations were carried out using a total of 71 EDTA whole blood, 69 EDTA plasma, 120

lithium-heparin plasma, and 72 serum samples. In an oral health interview, no participant reported any known current or past history of coronary artery disease, cardiac-releated medical condition, or other permanent medication. All the volunteers, with the exception of the 47 participants in the group assigned to lithium-heparin plasma collection, were also checked by echocardiography and the normal echocardiography was found in all the checked volunteers. The 99th percentile determination with lithium-heparin plasma was also reported previously in Euromedlab 2003 by us (the 99th percentile of the apparently healthy reference group was 0.025 $\mu$g/L ($n = 187$) [5]).

### 2.8. *Sample stability*

The sample stability studies were made with lithium-heparin whole blood, lithium-heparin plasma, EDTA whole blood, EDTA plasma, and serum samples. Samples were taken from a total of 10 patients suffering from acute MI (different durations of chest pain) or cardiac surgery. The samples were divided into three tubes. The tubes were kept at room temperature, at +4°C and at −20°C (not whole blood). The cTnI concentrations from the samples kept at room temperature were measured at 0.5 h, 1 h, 2 h, 2.5 h, 3 h, 5 h, 8 h, and 24 h after the first measurement. The first measurement was made about 30 minutes after the venipuncture (serum about 45 min). Sample stability at +4°C was determined at 1 h, 2 h, 3 h, 5 h, 8 h, and 24 h after the first measurement. The stabilities of the serum, lithium-heparin plasma, and EDTA plasma samples were determined by freezing and thawing the samples 3 times. These samples were kept at −20°C for at least 24 hours, and the cTnI concentrations were measured after the three freezing and thawing cycles. Before analysis, the frozen samples were thawed at room temperature, mixed, and centrifuged to remove any particulate material.

### 2.9. *Influence of hemodialysis and rheumatoid factor*

In these studies, we determined cTnI in 48 chronic renal failure patients before and after hemodialysis. The time between the two determinations was about 4-5 hours. We determined the cTnI concentrations in serum samples from 15 patients with positive rheumatoid factor but with no indication of myocardial infarction. cTnI was determined with the Innotrac Aio! and AxSYM analyzers in both studies.

### 2.10. *cTnI profiles of patients*

cTnI concentrations were determined on admission and at different time points after admission from 7 patients suffering from acute MI, 5 coronary artery bypass grafting (CABG) patients, 1 myocarditis patient, and 2 patients without a diagnosis of acute MI, of whom one had collapsus, chronic bronchitis, and liver tumour and the other spinal surgery and arrhythmias. The cTnI profiles of these samples were determined with Innotrac Aio!, Beckman Access, and Abbott AxSYM. Additionally, the cTnI profiles of 12 patients with acute MI, 9 patients with CABG, 1 with bypass surgery, and 1 with cardiomyopathy obtained with Innotrac Aio! and AxSYM were compared.

Table 1: Within-assay and interassay precision.

| $n = 20$ | Within-assay precision Mean $\mu$g/L | CV% |
|---|---|---|
| Plasma sample 1 | 0.037 | 7.2 |
| Plasma sample 2 | 0.30 | 3.2 |
| Plasma sample 3 | 3.7 | 5.0 |
| Whole blood 1 | 0.023 | 10.9 |
| Whole blood 2 | 0.12 | 3.4 |
| Whole blood 3 | 2.8 | 3.7 |
| | Interassay precision Mean $\mu$g/L | CV% |
| Quantimetrix Cardiasure 1 ($n = 40$) | 0.37 | 7.1 |
| Quantimetrix Cardiasure 2 ($n = 40$) | 1.3 | 4.2 |
| Quantimetrix Cardiasure 3 ($n = 40$) | 3.3 | 3.7 |
| Serum pool ($n = 40$) | 0.11 | 4.0 |
| Plasma pool 1 ($n = 30$) | 0.037 | 12.0 |
| Plasma pool 2 ($n = 30$) | 0.020 | 17.2 |

### 3. RESULTS

### 3.1. *Analytical performance*

The analytic detection limit of the assay was 0.007 $\mu$g/L. The precision data for pooled plasma, serum, and commercial controls are shown summarized in Table 1. Within-assay CVs were 3.2%–10.9%, and between-assay precision ranged from 4.0% to 17.2%. The functional sensitivity (CV = 20%) and the concentrate giving CV of 10% were approximately 0.02 and 0.04 $\mu$g/L, respectively, according to the precision results shown in Table 1 for patient pools. The cTnI concentration corresponding to a 10% CV and the detection limit obtained nearly confirmed the earlier published data by Pagani et al. [6].

The linearity in the tested range of 0.063–112 $\mu$g/L for cTnI was within acceptable limits. The recoveries for the different dilutions were 74%–107%.

Figure 1 shows the differences between the compared methods, expressed as Deming regression curves, percentage of the average, and plotted against the method average. The following correlations emerged between the assays: Innotrac Aio! = 0.45 × Access + 0.03, $R^2 = 0.947$ ($n = 91$, range < 20 $\mu$g/L with Aio!), Innotrac Aio! = 0.38 × Access + 0.01, $R^2 = 0.784$ ($n = 55$, range < 0.5 $\mu$g/L with Aio!), Innotrac Aio! = 0.06 × AxSYM + 0.11, $R^2 = 0.966$ ($n = 91$, range < 20 $\mu$g/L with Aio!), and Innotrac Aio! = 0.05 × AxSYM − 0.01, $R^2 = 0.588$ ($n = 55$, range < 0.5 $\mu$g/L with Aio!). The mean differences (95% confidence interval) for Aio! and Access cTnI and for Aio! and AxSYM cTnI were 78% and 155%, respectively.

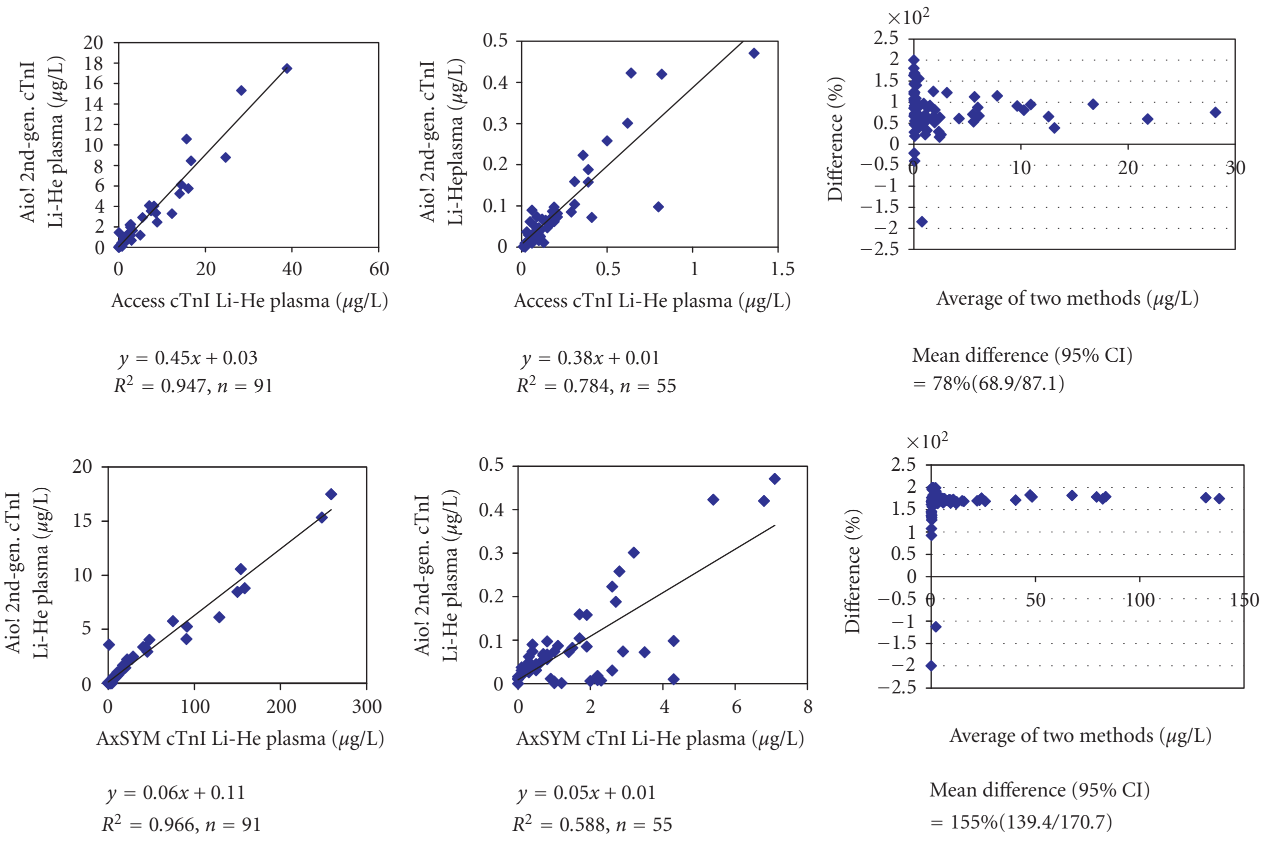

Figure 1: Correlation between Access Li-heparin plasma and Innotrac Aio! second-generation cTnI Li-heparin plasma, and AxSYM Li-heparin plasma and Innotrac Aio! second-generation cTnI Li-heparin plasma evaluated as Deming regression and plots of differences. Percentage of difference ($y$-axis) = [method A-method B/average of the two methods] × 100. CI = confidence interval.

The 99th percentiles of the healthy reference group with different sample matrices were all ≤ 0.03 μg/L. These results confirm our previous findings and Pagani et al.'s results suggesting that the 99th percentile limit could be at 0.03 μg/L [5, 6].

The analytical characteristics of cardiac troponin as reported by the manufacturers are 0.04 for Access and 0.3 μg/L for AxSYM as 99th percentiles, while the CVs of 10% are 0.06 for Access and 0.8 μg/L for AxSYM. Based on the 99th percentiles as a threshold value of the Access, AxSYM, and Innotrac Aio! second-generation cTnI assays, 14 samples from patients (from the total of 91, the clinical background was checked from all of them) were classified differently into following diagnostic categories: acute myocardial infarction, unstable angina pectoris, prolonged angina pectoris, non-ischemic chest pain, dyspnoe, coronary procedures (PTCA, CABG), congestive hearth failure, and others (infections, abdominal pain, pulmonary embolism, collapsus). When the CV of 10% was used as a threshold value, 29 patients were classified differently. In the second comparative study ($n = 97$), discrepancies between the results obtained by using the 99th percentile limit and the 10% total CV concentration limit in the AxSYM, Innotrac Aio! first- and second-generation cTnI assays were in seen in 8 samples and 17 samples, respectively. These patient groups were too limited to allow any detailed conclusions regarding diagnostics sensitivity and specificity but allow conclusions in a broader sense as to relative diagnostic performance of the compared assays. The second-generation Aio! assay was found to be superior to first-generation assay.

The sample matrix correlation study using lithium-heparin whole blood, lithium-heparin plasma, and serum samples yielded the following results: the slopes were lithium-heparin plasma = 0.90×lithium-heparin whole blood−0.02, $R^2 = 0.998$ and serum = 1.08 × lithium-heparin plasma − 0.01, $R^2 = 0.997$.

### 3.2. Sample stability

The level of cTnI in the serum, lithium-heparin plasma, and lithium-heparin whole blood samples ($n = 10$) was found to decrease from the original concentration less than 10% in 24 hours at room temperature, except at one time point, 3 hours, where the cTnI level in whole blood showed a 13% decrease. The cTnI level in EDTA whole blood and EDTA plasma decreased less than 10% in up to 1 hour and 1.5 hours, respectively. cTnI concentration at +4°C decreased less than 10% in all the matrices in 24 hours, except in EDTA

whole blood. Analyte concentration at −20°C decreased less than 10% in serum and lithium-heparin plasma, when freezing and thawing cycles were done, but cTnI level in EDTA plasma decreased more than 10% after one freeze-thaw cycle.

### 3.3. *Influence of hemodialysis or rheumatoid factor*

The study group consisted of 48 patients treated with hemodialysis (22 men and 26 women). The mean age of the patients was 57 years (range: 15–89 years). Samples were obtained before and after hemodialysis ($n = 96$). cTnI was measured from EDTA plasma samples with the Innotrac Aio! and AxSYM analyzers. The concentration ranges were 0.000–0.039 μg/L and 0.0–1.1 with the Aio! and AxSYM cTnI assays, respectively.

In the hemodialysis study, a total of 3 patient samples were above the 99th percentile limit (0.039, 0.040, and 0.035 μg/L), and only one patient had a plasma level above that cutoff limit at both measurements (0.039 and 0.040 μg/L). During hemodialysis session the cTnI values of 21/48 patients (43%) increased, while no change in cTnI values was seen in 15/48 patients (32%), and cTnI values of 12/48 patients (25%) decreased. However, the changes were not significant and may be due to the higher precision near the detection limit and to the background signal. Additionally, 2 of the samples were at the cutoff limit.

Altogether, 5 samples were above the 99th percentile limit (0.9, 0.9, 1.1, 0.5, and 0.6 μg/L), and two patients had both of these values above the cutoff limit (0.9/0.9 and 0.5/0.6 μg/L) using AxSYM assay. Additionally, 5 of the samples had concentration values at the cutoff. During hemodialysis session 4/48 patients (8%) had increase in cTnI values, 7/48 patients (15%) showed no change, and 37/48 patients (77%) had decrease in cTnI values. In conclusion, based on these results, the positive cTnI results appear to be due to the coronary artery disease of other potential injuries to the heart and not due to the hemodialysis. Additionally, the imprecision of > 10% CV at the 99th percentile of the reference group does not permit reliable determination of cTnI at this concentration with these methods.

False-positive troponin I values due to the presence of rheumatoid factor have been described [7]. We studied serum samples from 15 rheumatoid patients. The rheumatoid factors (RFs) measured using Hitachi 911 varied from 20 to 3630 U/L. Only one of the samples had a cTnI concentration (0.070 μg/L) above the 99th percentile with a very high RF concentration of 3630 U/L. This sample needs to be further investigated. The concentrations of cTnI in serum samples were also measured using the AxSYM analyzer. No measurable cTnI was observed in these samples with this analyzer.

### 3.4. *Differences in cTnI profiles between the methods*

The total of 38 patient profiles with different cardiac events using different commercial methods were determined. The profiles were similar, but in the 2/3 comparisons with

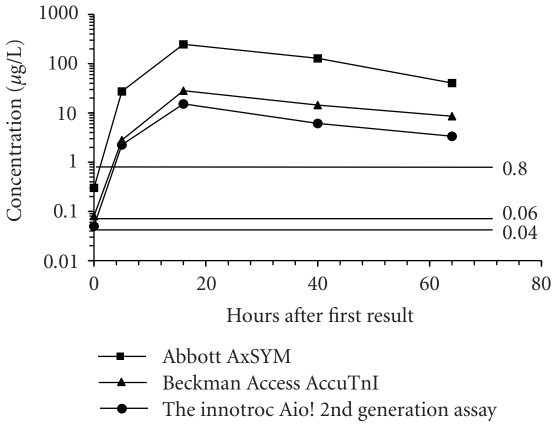

Figure 2: An example of cTnI profiles of patients with acute MI. The Innotrac Aio! second-generation assay is indicated as a line with fulled circles, Abbott AxSYM as a line with boxes, and Beckman Access AccuTnI as a line with triangles. The 10% total CV concentration limits (μg/L) of the methods are marked as horizontal lines.

second-generation Innotrac Aio!, AxSYM, and Access methods, the second-generation Innotrac Aio! and Access assays detected the released cTnI and myocardial damage earlier than the AxSYM assay. Figure 2 shows an example of the AMI patient's profile using the Innotrac Aio! second-generation assay in comparison with the commercial assays.

### 4. DISCUSSION

The complex molecular nature of cTnI complicates measurement by immunoassays, causing the commercial assays to detect cTnI differently. The differences between the results can be explained by the use of different antibodies in the assays, the lack of international standardization and heterophilic antibodies, which may cause false-positive results in immunoassays. The different distributions of cTnI forms in the early versus late phase of myocardial infarction may explain the differences. It has been shown that, in addition to occuring as free cTnI and in binary complexes with TnI and TnT, TnI may exist in phosphorylated, oxidized, and proteolytically degraded forms [8]. They can change the structure and conformation of the molecule and thereby affect the antibody- antigen interaction [9]. It has also been shown that troponin is released into the bloodstream of patients with acute myocardial infarction not in a free form but as a complex [10]. The antibodies used in the assays should ideally recognize equally both free and complexed forms. It has been recommended that the antibodies used for the development of reliable cardiac troponin assays should preferably recognize epitopes that are located in the stable part of the molecule and are not affected by complex formation and other *in vivo* modifications [11].

Previously, Eriksson et al. [12] evaluated the presence of interfering factors in cTnI assays by measuring the recovery of cTnI added to samples from volunteers and from patients with acute coronary syndromes. The factor may cause variable inhibition of cTnI immunoreactivity, from mild to very severe, depending on the amount of interfering factor in the sample. The interfering factor had the greatest effect on cTnI measurements when there were only small amounts of cTnI present in the sample, as in the early hours of an MI or unstable angina event. Eriksson et al. proposed as a solution to the problem a multi-antibody assay, in which the antibody combinations are chosen from the terminal parts with a mid-fragment part, because the most interference-free antibodies are those against epitopes in the terminal parts of the molecule, which are not present in the fragmented cTnI molecules. According to the manufacturer, the Innotrac Aio! second-generation assay has been developed using this information. This may explain the earlier detection of released cTnI and myocardial damage compared to the AxSYM assay as seen in Figure 2. The Access assay seemed to detect the cTnI of MI patients equally early as the Aio! assay, thus showing that the antibodies may have been chosen from the same regions. The clearly improved performance of the Aio! second-generation assay compared to the first-generation assay near the cutoff limit could be explained partly by the new antibodies used in the assay. The lack of reproducibility (CV% > 10) by AxSYM at very low concentrations could also explain the lack of correlation between the assays at low levels [13, 14]. On the other hand, Abbott has recently introduced a second-generation cTnI assay using AxSYM analyzer.

The definition of MI by international cardiology associations led to a further reduction of the cutoff values. To avoid misclassification arising from assay imprecision, the consensus committee proposed that the 99th percentile reference limit should be measured with total imprecision (CV) < 10%. The imprecision of the Innotrac Aio! second-generation assay was highly superior to that of the previously published first-generation assay [15]. In our precision study, the ratio 10% CV/99th was 1.3, proving the good imprecision of the assay.

Plasma has been recommended as the specimen of choice by both the cardiology and the laboratory medicine communities, to keep the turnaround time short [11]. However, there may be significant differences between serum and plasma concentrations of troponins when different analytical systems are used [16]. According to our matrix comparison data, lithium-heparin plasma, whole blood, and serum are all acceptable matrices for the determination of cTnI with the Aio! second-generation assay.

This study was a preliminary study showing the characteristics of a new second-generation assay of cTnI on the Innotrac Aio! Analyzer. To improve the statistics the number of patients and controls should be higher.

The ability to work with whole blood samples provides a great advantage, saving time and work, and if the follow-up testing is carried out at a central laboratory, it is possible to analyze serum and plasma by using the same system. Innotrac Aio! is quick and easy to use and maintain, and thus suitable for use in emergency rooms, coronary care units, satellite laboratories, and central laboratories.

### ACKNOWLEDGMENTS

We thank Innotrac Diagnostics for providing the Aio! system and reagents and for technical assistance and we especially thank Dr. Jussi Kurittu of Innotrac Diagnostics. We also thank Ordior Oy, for providing the Access cTnI reagents for comparative studies. The technical assistance of the Oulu University Hospital Laboratory staff is greatly appreciated.

### REFERENCES

[1] The Joint European Society of Cardiology/ American College of Cardiology Committee, "Myocardial infarction redefined—a consensus document of The Joint European Society of Cardiology/American College of Cardiology committee for the redefinition of myocardial infarction," *Journal of the American College of Cardiology*, vol. 36, no. 3, pp. 959–969, 2000.

[2] "ACC/AHA guidelines for the management of patients with unstable angina and non-ST segment elevation myocardial infarction: executive summary and recommendations. A report of the American College of Cardiology/ American Heart Association Task Force on Practice Guidelines (Committee on the Management of Patients with Unstable Angina)," *Circulation*, vol. 102, pp. 1893–1900, 2000.

[3] K. Petterson, T. Katajamäki, K. Irjala, V. Leppänen, K. Majamaa-Voltti, and P. Laitinen, "Time-resolved fluorometry (TRF)-based immunoassay concept for rapid and quantitative determination of biochemical myocardial infarction markers from whole blood, serum and plasma," *Luminescence*, vol. 15, no. 6, pp. 399–407, 2000.

[4] T. Lövren, L. Meriö, K. Mitrunen, et al., "One-step all-in-one dry reagent immunoassays with fluorescent Europium chelate label and time-resolved fluorometry," *Clinical Chemistry*, vol. 42, no. 8 pt 1, pp. 1196–1201, 1996.

[5] P. Hedberg, J. Kurittu, and M. Puukka, "Evaluation of the new Innotrac Aio! $2^{nd}$ generation cardiac troponin I assay," *Clinical Chemistry and Laboratory Medicine*, vol. 41, no. supplement W-125, p. S426, 2003.

[6] F. Pagani, F. Stefini, and M. Panteghini, "Innotrac Aio! second-generation cardiac troponin I assay: Imprecision profile and other key characteristics for clinical use," *Clinical Chemistry*, vol. 50, pp. 1271–1272, 2004.

[7] A. Dasgupta, S. Banerjee, and P. Datta, "False-positive troponin I in the MEIA due to the presence of rheumatoid factors in serum. Elimination of this interference by using a polyclonal antisera against rheumatoid factors," *American Journal of Clinical Pathology*, vol. 112, no. 6, pp. 753–756, 1999.

[8] A. Katrukha, A. Bereznikova, V. Filatov, et al., "Degradation of cardiac troponin I: implication for reliable immunodetection," *Clinical Chemistry*, vol. 44, no. 12, pp. 2433–2440, 1998.

[9] A. Katrukha, A. Bereznikova, V. Filatov, and T. Esakova, "Biochemical factors influencing measurement of cardiac troponin I in serum (Review)," *Clinical Chemistry and Laboratory Medicine*, vol. 37, no. 11-12, pp. 1091–1095, 1999.

[10] A. Katrukha, A. Bereznikova, T. Esakova, et al., "Troponin I is released in bloodstream of patients with acute myocardial infarction not in free form but as complex," *Clinical Chemistry*, vol. 43, pp. 1379–1385, 1997.

[11] M. Panteghini, W. Gerhardt, F. S. Apple, F. Dati, J. Ravkilde, and A. H. Wu, "Quality specifications for cardiac troponin assays," *Clinical Chemistry and Laboratory Medicine*, vol. 39, no. 2, pp. 175–179, 2001.

[12] S. Eriksson, M. Junikka, P. Laitinen, K. Majamaa-Voltti, H. Alfthan, and K. Pettersson, “Negative interference in cardiac troponin I immunoassays from a frequently occuring serum and plasma component,” *Clinical Chemistry*, vol. 49, no. 7, pp. 1095–1104, 2003.

[13] Z. Zaman, S. De Spiegeleer, M. Gerits, and N. Blanckaert, “Analytical and clinical performance of two cardiac troponin I immunoassays,” *Clinical Chemistry and Laboratory Medicine*, vol. 37, no. 9, pp. 889–897, 1999.

[14] K. S. Quinn-Hall, S. W. Bateman, A. H. Wu, S. Wieczorek, G. A. Ficher, and K. T. Yeo, “Functional sensitivity of cardiac troponin assays and implications for risk stratification (Absract),” *Clinical Chemistry*, vol. 46, no. supplement 6, p. A78, 2000.

[15] P. Hedberg, J. Valkama, and M. Puukka, “Analytical performance of time-resolved fluorometry-based Innotrac Aio! cardiac marker immunoassays,” *Scandinavian Journal of Clinical and Laboratory Investigation*, vol. 63, no. 1, pp. 55–64, 2003.

[16] W. Gerhardt, G. Nordin, A. Herbert, et al., “Troponin T and I assays show decreased concentrations in heparin plasma compared with serum: Lower recoveries in early than in late phase of myocardial injury,” *Clinical Chemistry*, vol. 46, pp. 817–821, 2000.

